# Supplementary material for: The causal role of circulating inflammatory markers in osteoporosis: a bidirectional Mendelian randomized study
Source: Front Immunol. 2024 Jul 18;15:1412298. doi: 10.3389/fimmu.2024.1412298 (PMC11291241; doi:10.3389/fimmu.2024.1412298)
Supplement: Supplementary file 1 [file DataSheet_1.zip › supplementary material/OSTEOPOROSIS-CIM/GCST90274818.tsv.gz_leave_one_out.pdf]

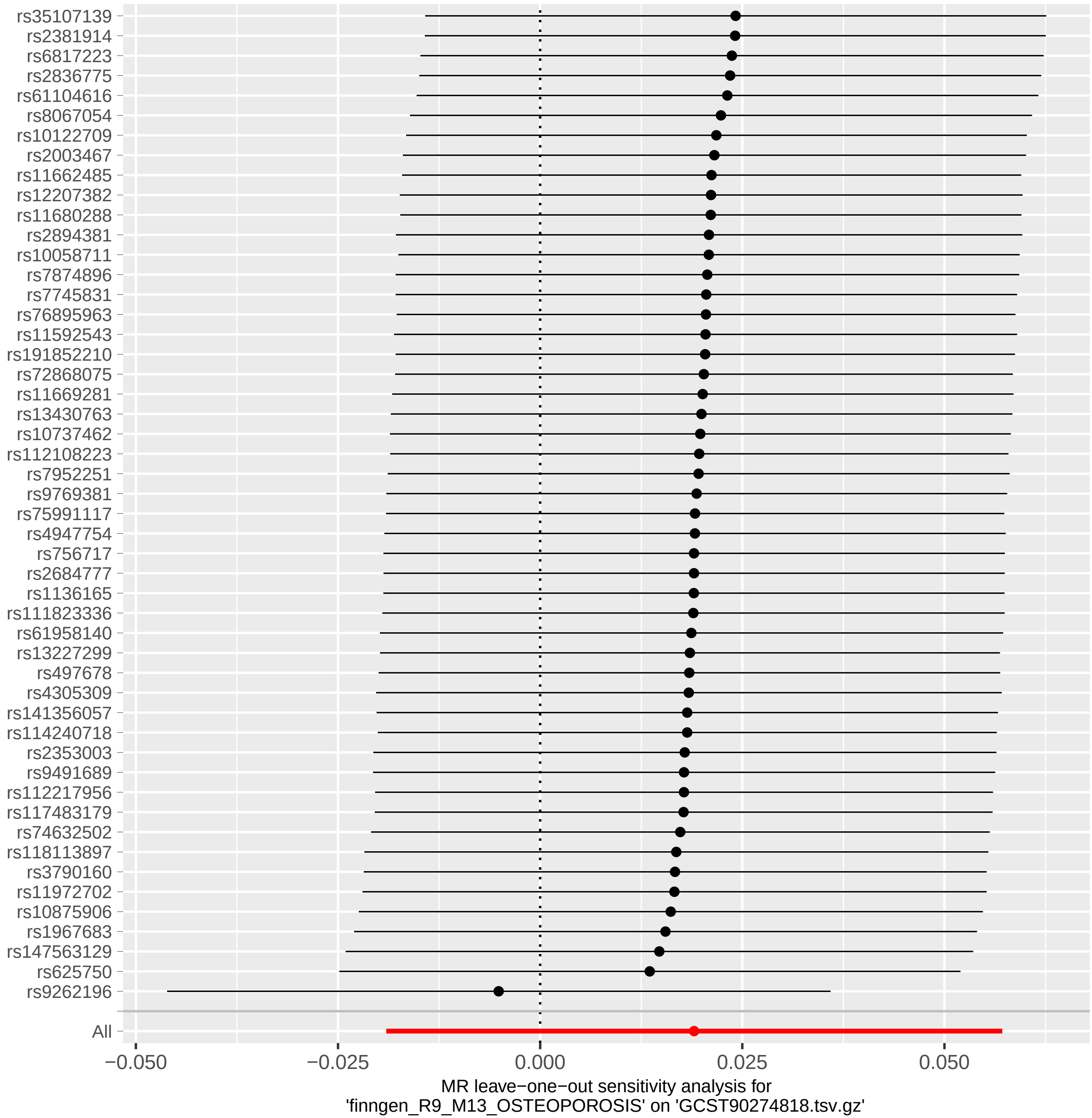
rs35107139
rs2381914
rs6817223
rs2836775
rs61104616
rs8067054
rs10122709
rs2003467
rs11662485
rs12207382
rs11680288
rs2894381
rs10058711
rs7874896
rs7745831
rs76895963
rs11592543
rs191852210
rs72868075
rs11669281
rs13430763
rs10737462
rs112108223
rs7952251
rs9769381
rs75991117
rs4947754
rs756717
rs2684777
rs1136165
rs111823336
rs61958140
rs13227299
rs497678
rs4305309
rs141356057
rs114240718
rs2353003
rs9491689
rs112217956
rs117483179
rs74632502
rs118113897
rs3790160
rs11972702
rs10875906
rs1967683
rs147563129
rs625750
rs9262196
All
−0.050
−0.025
0.000
0.025
0.050
MR leave−one−out sensitivity analysis for
'finngen_R9_M13_OSTEOPOROSIS' on 'GCST90274818.tsv.gz'
